# Improved Variational Mode Decomposition Based on Scale Space Representation for Fault Diagnosis of Rolling Bearings

**DOI:** 10.3390/s25113542

**Published:** 2025-06-04

**Authors:** Baoxiang Wang, Guoqing Liu, Jihai Dai, Chuancang Ding

**Affiliations:** 1 School of Mechanical Engineering, Suzhou University of Science and Technology, Suzhou 215009, China; 2 School of Rail Transportation, Intelligent Urban Rail Engineering Research Center of Jiangsu Province, Soochow University, Suzhou 215131, China; 3 State Key Laboratory of Mechanical Transmission for Advanced Equipment, Chongqing University, Chongqing 400044, China

**Keywords:** rolling bearings, improved variational mode decomposition, scale space representation, multipoint kurtosis, fault diagnosis, vibration analysis

## Abstract

Accurate extraction of weak fault information from non-stationary vibration signals collected by vibration sensors is challenging due to severe noise and interference. While variational mode decomposition (VMD) has been effective in fault diagnosis, its reliance on predefined parameters, such as center frequencies and mode number, limits its adaptability and performance across different signal characteristics. To address these limitations, this paper proposes an improved variational mode decomposition (IVMD) method that enhances diagnostic performance by adaptively determining key parameters based on scale space representation. In concrete, the approach constructs a scale space by computing the inner product between the signal’s Fourier spectrum and a Gaussian function, and then identifies both the mode number and initial center frequencies through peak detection, ensuring more accurate and stable decomposition. Moreover, a multipoint kurtosis (MKurt) criterion is further employed to identify fault-relevant components, which are then merged to suppress redundancy and enhance diagnostic clarity. Experimental validation on locomotive bearings with inner race faults and compound faults demonstrates that IVMD outperforms conventional VMD by effectively extracting fault features obscured by noise. The results confirm the robustness and adaptability of IVMD, making it a promising tool for fault diagnosis in complex industrial environments.

## 1. Introduction

Rolling element bearings (REBs) are essential components in rotating machinery, commonly used in industries such as manufacturing and energy [1,2,3]. These bearings are subject to heavy operational loads and non-stationary working conditions, which often result in wear and failure over time, leading to costly downtime and significant economic losses. As a result, fault diagnosis is crucial for maintaining equipment reliability and operational safety [4]. Among various monitoring techniques, vibration signal analysis has emerged as the most widely adopted method due to its sensitivity to mechanical faults and its ability to capture dynamic system responses [5,6].

When a fault occurs in a rolling element bearing, periodic impacts excite the structural resonances of the bearing and adjacent components, generating transient impulses that manifest at characteristic frequencies. However, due to the complexity of mechanical structures and harsh operational environments, these fault impulses are often obscured by significant background noise and other interferences, making fault diagnosis particularly challenging [7]. The accurate filtering of noise and extraction of meaningful fault features remain critical challenges in vibration-based fault detection. Over the past few decades, various methods have been developed to address this problem, including wavelet transform (WT) [8,9,10], empirical mode decomposition (EMD) [11,12], local mean decomposition (LMD) [13,14] and singular value decomposition (SVD) [15,16], spectral kurtosis (SK) [17,18] and variational mode decomposition (VMD), etc.

Among these, VMD has gained notable attention due to its ability to decompose complex signals into a set of band-limited, quasi-orthogonal intrinsic mode functions (IMFs). Unlike EMD or LMD, which suffer from a lack of theoretical rigor, VMD formulates the decomposition as a constrained variational problem, enabling better mode separation and noise resilience. These advantages have led to its successful application in various domains, including machinery fault detection, signal denoising, and condition monitoring. For instance, Wang et al. [19] investigated the equivalent filtering characteristics of VMD and applied it to detect multiple rubbing-caused signatures for rotor-stator fault diagnosis. Zhang et al. [20] proposed a hybrid fault diagnosis approach based on VMD and total variation minimization for bearing fault diagnosis. Moreover, Zhang et al. [21] applied VMD to diagnose faults in bearings of a multistage centrifugal pump. Nevertheless, the accuracy and effectiveness of VMD are highly sensitive to the selection of key parameters: the number of modes and the quadratic penalty term, which governs bandwidth constraints. Typically, these parameters are manually set based on empirical knowledge, limiting the method’s adaptability and potentially yielding suboptimal decomposition. To address this, researchers have proposed various strategies for parameter optimization. Li et al. [22] introduced an independence-oriented VMD method that uses peak searching and similarity principles to determine the most suitable mode number, although it does not account for the impact of the bandwidth control parameter. Zhang et al. [23] presented a parameter-adaptive VMD method using the grasshopper optimization algorithm (GOA) to estimate the optimal mode number and bandwidth control parameter adaptively. Lian et al. [24] proposed an adaptive VMD that automatically determines the mode number based on the characteristics of the intrinsic mode functions.

Although several improved VMD approaches have been proposed, most focus on optimizing parameters such as the mode number and penalty factor using optimization algorithms, which are often time consuming and complex to implement. Moreover, the influence of the initial center frequencies $w_{k}^{0}$—an essential factor for stable and accurate decomposition—has been largely overlooked. Since the selection of key parameters in VMD strongly influences its decomposition performance and diagnostic reliability, a fully adaptive strategy is essential. To this end, this paper proposes an Improved Variational Mode Decomposition (IVMD) method that incorporates a scale space representation to adaptively determine both the initial center frequencies and the number of modes in a data-driven manner. In addition, multipoint kurtosis (MKurt) is introduced to guide the selection and merging of modes, ensuring that critical fault features are preserved. Specifically, the IVMD method begins by generating the scale space representation through the inner product between the signal’s Fourier spectrum and a Gaussian function. The initial center frequencies are then determined based on the local maxima in the scale space. Once these parameters are established, the signal is decomposed into subcomponents using VMD. Subcomponents with large MKurt that contain fault-related features are merged to improve fault detection. Experimental results from bearings with various faults demonstrate that IVMD effectively extracts fault features, even in the presence of significant noise and interference. By adaptively selecting key parameters, IVMD outperforms conventional VMD, making it a robust and reliable tool for fault diagnosis in rolling element bearings.

The main contributions of this work can be summarized as follows:

(1) A novel IVMD framework is proposed, which employs scale space representation to adaptively determine both the number of modes and their initial center frequencies, thereby overcoming the reliance on manually set parameters in conventional VMD.
(2) The MKurt-based mode merging strategy is introduced to enhance fault-relevant components and suppress redundant modes, improving the accuracy of impulse extraction in noisy environments.
(3) Experimental validation on locomotive bearings with both single and compound faults demonstrates that the proposed method significantly outperforms conventional VMD in fault detection capability.

The remainder of this paper is organized as follows. Section 2 provides a comprehensive review of VMD. Section 3 introduces the challenges associated with parameter selection, such as mode number and center frequency initialization. Section 4 details the proposed IVMD based on scale space representation. Section 5 validates the effectiveness of the proposed IVMD through experimental studies, including comparisons with traditional VMD using vibration signals from bearings with compound faults. Finally, Section 6 concludes the paper.

## 2. Theory of Variational Mode Decomposition

The VMD is a signal processing technique that decomposes a given signal into a set of band-limited sub-components or modes *u_k_*, each centered around a specific frequency, while preserving the ability to reconstruct the original signal [25]. The decomposition is formulated as a constrained variational problem aimed at minimizing the sum of bandwidths of all modes under the constraint that their sum reconstructs the original signal:

(1) $$\begin{array}{l} \underset{\left\{u_{k}\},\{w_{k}\right\}}{\min}\left\{\sum_{k}{\left\|\partial_{t}\left[\left(\delta \left(t\right)+\frac{j}{\pi t}\right)*u_{k}\left(t\right)\right]e^{-jw_{k}t}\right\|}_{2}^{2}\right\}\\ \mathrm{s.t.}\sum_{k}u_{k}=x \end{array}$$

where $\left\{u_{k}\}=\{u_{1},\;u_{2},\;\ldots ,\;u_{K}\}\;and\;\{w_{k}\}=\{w_{1},\;w_{2},\;\ldots ,\;w_{K}\right\}$ are the modes and the center frequencies, respectively. *K* is the number of decomposed modes. In (1), the problem consists of three key steps. First, Hilbert Transform is applied to obtain the analytic signal and compute the unilateral frequency spectrum of each mode *u_k_*; second, frequency shifting is conducted by demodulating the spectrum to the baseband via multiplication with an exponential function tuned to the estimated center frequency *w_k_*; third, bandwidth estimation is achieved by computing the squared *L*_2_-norm of the gradient of the demodulated signal.

To solve the constrained variational problem in (1), it is converted into an unconstrained optimization form using the augmented Lagrangian method. This involves introducing a quadratic penalty term *α* and Lagrangian multiplier *λ*(*t*), resulting in the following formulation:

(2) $$\begin{array}{l} L\left(\left\{u_{k}\right\},\left\{w_{k}\right\},\lambda \right)\\ =\alpha \cdot {\sum_{k}\left\|\partial_{t}\left[\left(\delta \left(t\right)+\frac{j}{\pi t}\right)*u_{k}\left(t\right)\right]e^{-jw_{k}t}\right\|}_{2}^{2}\\ +{\left\|f\left(t\right)-\sum_{k}u_{k}\left(t\right)\right\|}_{2}^{2}+\langle \lambda \left(t\right),f\left(t\right)-\sum_{k}u_{k}\left(t\right)\rangle \end{array}$$

The optimization problem in Equation (2) is then solved using the alternating direction method of multipliers (ADMM). The entire solving process of VMD is summarized in Algorithm 1, which iteratively updates $u_{k}^{n+1}$, $w_{k}^{n+1}$ and $\lambda_{k}^{n+1}$, until a convergence condition defined by threshold *c* is met.
**Algorithm 1**: Variational Mode DecompositionInitialize $\left\{u_{k}^{0}\right\},\left\{w_{k}^{0}\right\},\lambda^{0},n=0$
**repeat****for** *k* = 1 to *K*
**do**
    Update $u_{k}$: $u_{k}^{n+1}=\underset{u_{k}}{\arg\min}L\left(\left\{u_{i<k}^{n+1}\right\},\left\{u_{i\geq k}^{n}\right\},\left\{w_{i}^{n}\right\},\lambda^{n}\right)$
Update $w_{k}$: $w_{k}^{n+1}=\underset{w_{k}}{\arg\min}L\left(\left\{u_{i}^{n+1}\right\},\left\{w_{i<k}^{n+1}\right\},\left\{w_{i\geq k}^{n}\right\},\lambda^{n}\right)$
Update $\lambda^{n}$: $\lambda^{n+1}=\lambda^{n}+\tau \left(f-\sum_{k}u_{k}^{n+1}\right)$
**end for**    
n←n+1
**until** convergence: $\sum_{k}\left[{\left\|u_{k}^{n+1}-u_{k}^{n}\right\|}_{2}^{2}/{\left\|u_{k}^{n}\right\|}_{2}^{2}\right]<c$

For the minimization problems of $u_{k}^{n+1}$, $w_{k}^{n+1}$ in Algorithm 1, the solution for the intrinsic modes $\left\{u_{k}\right\}$ can be obtained by Wiener filtering in Fourier domain [26], and the center frequency $\left\{w_{k}\right\}$ can be updated as the center of gravity of the mode’s power spectrum. Specifically, the mode ${\hat{u}}_{k}^{n+1}(w)$ and center frequency ${\hat{w}}_{k}^{n+1}$ can be represented by

(3) $${\hat{u}}_{k}^{n+1}(w)=\frac{\hat{f}(w)-\sum_{i\neq k}{\hat{u}}_{i}(w)+\frac{\hat{\lambda }(w)}{2}}{1+2\alpha {\left(w-w_{k}\right)}^{2}}$$

(4) $$w_{k}^{n+1}=\frac{\int_{0}^{\infty }w{\left|{\hat{u}}_{k}(w)\right|}^{2}dw}{\int_{0}^{\infty }{\left|{\hat{u}}_{k}(w)\right|}^{2}dw}$$

From the above descriptions, there are several parameters that need to be specified in advance. They are the mode number *K*, initial center frequencies $w_{k}^{0}$, the quadratic penalty term *α*, the noise-tolerance *τ* and the tolerance of convergence criterion *c*. Based on prior studies [22,23,27], it has been demonstrated that the values of *τ* and *c* exert minimal influence on the decomposition performance. Therefore, the default settings in the original VMD algorithm are adopted, with *τ* = 0 and *c* = 1 × 10^−6^. For vibration signals, a penalty term *α* = 2000 has proven effective.

Notably, initial center frequencies $w_{k}^{0}$ of all *K* modes, should be selected carefully based on the application scenario and significantly influence the decomposition results.

## 3. Motivation of the Proposed IVMD

To illustrate the impact of key parameters, namely the initial center frequencies $w_{k}^{0}$ and mode number *K*, on the decomposition performance of VMD, a simulated vibration signal containing compound bearing faults, specifically inner race and outer race defects, is constructed as follows [28]:

(5) $$\begin{array}{l} x(t)\\ =\sum_{m}A_{m}\cos\left(2\pi f_{m}t+\alpha_{m}\right)+\sum_{i}D_{1}S_{1}(t-iT_{1}-\tau_{i})+\sum_{n}D_{2}S_{2}(t-nT_{2}-\tau_{n})+n\left(t\right) \end{array}$$

The first term simulates discrete harmonic interferences originating from rotors, shafts, gearboxes, or other rotating components, where *A_m_* and *α_m_* denote amplitude and initial phase, respectively. The second and third terms represent fault impulses generated by the inner race and outer race faults. Here, *D_i_* indicates impulse amplitude, and *T_d_* is the time interval between successive impulses. To simulate the slip phenomenon commonly observed in bearing vibrations, a random variable *τ_i_* with zero mean, typically accounting for 1–2% of the fault period, is introduced. *n*(*t*) represents additive Gaussian white noise, simulating environmental interference or sensor noise commonly present in real-world measurements.

When a rolling element strikes a surface defect, it excites structural resonance in the system, producing a sequence of impulses as rolling elements pass over the damaged area. The frequency of these impulses is uniquely determined by the rotational speed, fault location, and geometric configuration of the bearing. Notably, multiple faults usually excite distinct or overlapping resonant frequencies [28]. Generally, the defect impulses can generally be described as an exponentially decaying sinusoidal waveform:

(6) $$S_{i}=e^{-\xi t}\sin(2\pi f_{r}t)$$

where *f_r_* is the structural resonance frequency excited by the impact, and *ξ* is the decay rate.

**Figure 1:**
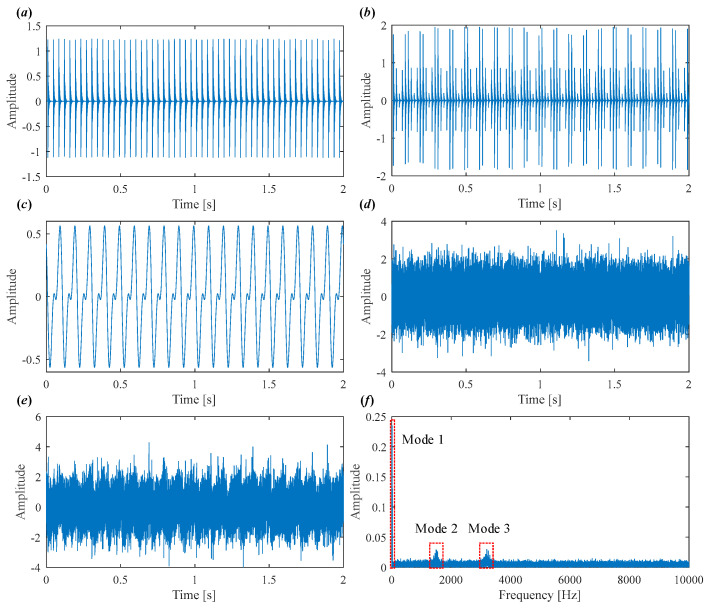
(**a**) Outer race fault impulses. (**b**) Inner race fault impulses. (**c**) Harmonics. (**d**) Gaussian noise. (**e**) Synthetic signal. (**f**) Frequency spectrum.

This signal model (5) effectively mimics the dynamic characteristics of real bearing fault signals, including overlapping impulse sequences, harmonic interference, and stochastic fluctuations, thereby providing a suitable case for validating the decomposition and fault feature extraction capabilities of the proposed IVMD. In this simulation, the outer race is fixed, and the inner race rotates with the shaft. Both outer and inner race faults are present, exciting two distinct resonance frequencies. The simulation parameters are summarized in Table 1, and waveform illustrations are provided in Figure 1. Figure 1a–d show outer race fault impulses, inner race fault impulses, harmonics, and Gaussian noise, respectively. The final synthetic signal is obtained by superimposing harmonic components, defect impulses, and Gaussian noise, achieving a signal-to-noise ratio (SNR) of −5 dB, as shown in Figure 1e. Meanwhile, the frequency spectrum of mixed signal is displayed in Figure 1f, from which the rotating frequency and two resonance frequency bands are obviously observed.

**Figure 2:**
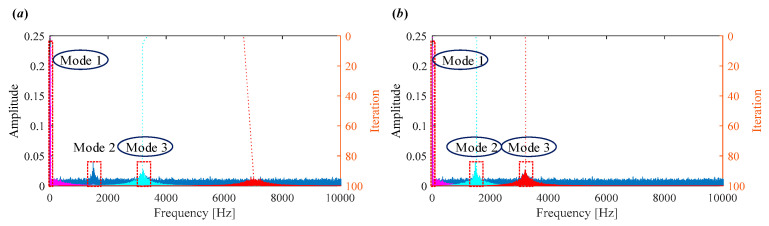
Iterative process of center frequencies in VMD when (**a**) $w_{k}^{0}=P_{u}$ (**b**) $w_{k}^{0}=\{0,1500,3200\}$.

The initialization of center frequencies $w_{k}^{0}$, *k* = 1, 2, …, *K* significantly influences the performance of VMD. Common strategies for setting $w_{k}^{0}$ include: uniformed spaced distribution $P_{u}$, zero initial $P_{z}$ and initialize randomly $P_{r}$ [29]. However, these fixed schemes are generally non-adaptive and may yield suboptimal decomposition results depending on the input signal. To demonstrate this, a simulation is performed with *α* = 2000 and *K* = 3. When $w_{k}^{0}$ is initialized using $P_{u}$, VMD fails to separate the components effectively (see Figure 2a). In contrast, when $w_{k}^{0}=\{0,\;1500,\;3200\}$, the modes are accurately located (Figure 2b). This highlights the fact that VMD does not guarantee convergence to a global minimum, and its results heavily depend on the initialization of center frequencies. The above analysis indicates the importance of proper center frequency selection. Thus, adaptive selection of $w_{k}^{0}$ remains a crucial yet challenging aspect in practical applications.

Moreover, to evaluate the effect of under- and over-estimating mode number *K*, the simulated signal is decomposed with different mode numbers ranging from 2 to 5, while keeping the quadratic penalty term *α* = 2000 and selecting $P_{u}$ as a prior initialization.

**Figure 3:**
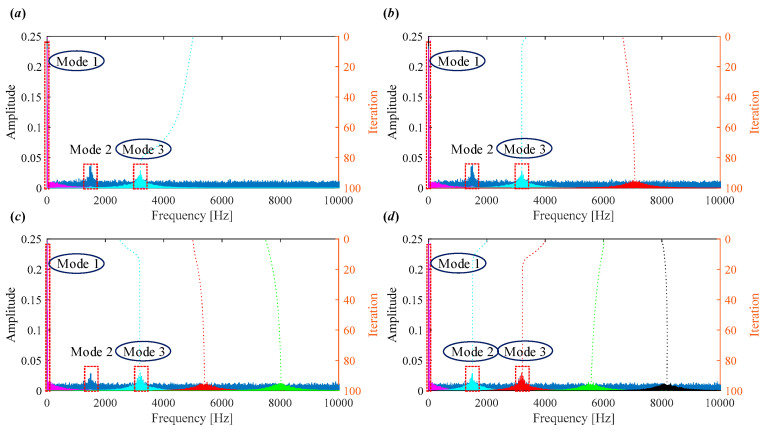
Iterative process of center frequencies in VMD when (**a**) *K* = 2; (**b**) *K* = 3; (**c**) *K* = 4; (**d**) *K* = 5.

The decomposition results when *K* = 2–5 are shown in Figure 3. When the number of modes *K* is underestimated (e.g., *K* = 2), one important component is lost, leading to incomplete decomposition (Figure 3a). With the optimal mode number selection (e.g., *K* = 3), three components are successfully extracted; however, they do not correspond to the three components of interest (Figure 3b). In the near-optimal case (*K* = 4), two components are accurately identified, but one remains indistinct (Figure 3c). When *K* is overestimated (e.g., *K* = 5), the three main components are still extracted; however, the additional modes primarily capture noise (Figure 3d).

These results suggest that both the number of modes *K* and the initial center frequencies $w_{k}^{0}$ play a pivotal role in determining the accuracy and quality of the decomposition. Therefore, careful selection of these parameters is essential for achieving optimal performance.

## 4. The Proposed IVMD for Bearing Fault Diagnosis

As previously discussed, the initialization of center frequencies $w_{k}^{0}$ of *K* modes is crucial for ensuring the effective performance of VMD. To address this issue, this paper introduces IVMD based on scale-space representation, which enables data-driven determination of the initial center frequencies. Meanwhile, a multipoint kurtosis-based mode merging strategy is also developed for fault feature enhancement. Together, these two aspects significantly improve the decomposition accuracy and robustness of the method, particularly in the context of bearing fault diagnosis.

### 4.1. Scale Space Representation Based Parameter Initialization

For a discrete-time vibration signal *x*(*t*), its discrete Fourier transform (DFT) *X*(*f*) is defined as

(7) $$X(f)=\sum_{t=-\infty }^{+\infty }x(t)e^{-j2\pi ft}$$

To construct the scale-space representation, a kernel function is introduced as $g\left(f,n\right)=\frac{1}{\sqrt{2\pi f}}e^{-\frac{n^{2}}{2f}}$, where *n* is called the scale parameter. The discrete scale space representation of the signal’s Fourier spectrum is then defined as

(8) $$L(f,n)=g(f,n)*X(f)=\sum_{-M}^{+M}g(\tau ,n)X(f-\tau )$$

where *M* must be sufficiently large to minimize the approximation error caused by truncation of the Gaussian kernel. A common choice is to set $M=C\sqrt{n}+1$ with 3≤C≤6. In this study, *C* = 6 is adopted to ensure the approximation error remains below $10^{-9}.$

To extract mechanical fault characteristic frequencies while ensuring that each decomposed sub-component retains fault-related information, the scale parameter should suppress characteristics below a predefined threshold $f_{ch}$, i.e., $\sqrt{n}\geq f_{ch}$ [30,31]. Since the fault characteristic frequency $f_{ch}$ and its harmonics provide strong evidence for localized defects, a relatively large-scale parameter is typically preferred. However, excessively large values may mask weak fault features. To balance resolution and sensitivity, the scale parameter is set as

(9) $$\sqrt{n}=\mu f_{ch}$$

where μ=2−4. The setting of μ is not restricted but suggested in this range. According to [32], the scale space representation *L*(*f*, *n*) becomes smoother with increasing *n*, and small fluctuations in *n* have minimal effect on the analysis results. Based on trial and error, $\sqrt{n}=3f_{ch}$ is selected.

**Figure 4:**
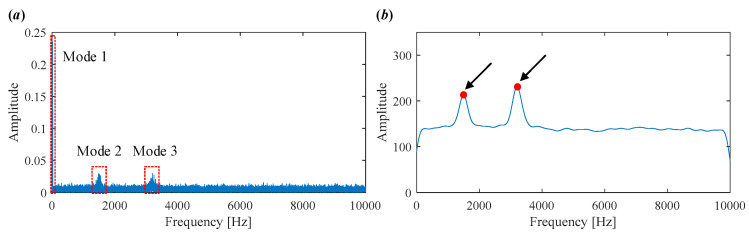
(**a**) Frequency spectrum of simulated signal. (**b**) Scale space representation.

The scale space representation of the simulated signal is shown in Figure 4. The scale-space representation involves smoothing the frequency spectrum at various scales, which helps in detecting prominent peaks corresponding to significant frequency components. By identifying these local maxima, one can effectively initialize the center frequencies for each mode in VMD, leading to improved decomposition performance. For example, in Figure 4, it is evident that the center frequencies corresponding to Mode 2 and Mode 3 are clearly identifiable.

However, low-frequency components falling below the $\sqrt{n}$ may be excluded from the initialization process. As shown in Figure 4, while the center frequencies for Mode 2 and Mode 3 are clearly captured, Mode 1, which corresponds to a low-frequency component, is initially omitted because its frequency falls below the detection threshold. To address this, we consistently assign the first center frequency as $w_{1}^{0}=0$. This manual setting is a general rule in our method, designed to ensure that low-frequency content—such as fundamental rotating frequencies and their harmonics—is retained. This consideration is particularly critical in applications like bearing fault diagnosis, where such low-frequency features often carry significant fault-related information. By including $w_{1}^{0}=0\;$ as part of the standard initialization, it enhances the completeness of the frequency decomposition and reduces the risk of missing diagnostically relevant components.

### 4.2. Multipoint Kurtosis-Based Mode Merging

Once the initial center frequencies are determined via scale-space representation, VMD is performed to decompose the signal into a set of intrinsic mode functions (IMFs), each represented as

(10) $$x(t)=\sum_{k=1}^{K}u_{k}(t)=\sum_{k}^{K}A_{k}(t)\cos(\phi_{k}(t))$$

where $A_{k}\left(t\right)$ and $\emptyset_{k}\left(t\right)$ are the instantaneous amplitude and phase of the *k*-th mode, respectively.

Given that multiple IMFs may contain similar or overlapping information, it is essential to eliminate redundancy by merging modes with analogous fault-related features. For this purpose, the multipoint kurtosis (MKurt) of the envelope spectrum is employed as a criterion. MKurt is defined as

(11) $$MKurt(\vec{y},\vec{t})=\frac{{\left(\sum_{n=1}^{N}t_{n}^{2}\right)}^{2}}{\sum_{n=1}^{N}t_{n}^{8}}\frac{\sum_{n=1}^{N}{\left(t_{n}y_{n}\right)}^{4}}{{\left(\sum_{n=1}^{N}y_{n}^{2}\right)}^{2}}$$

where *N* is the length, *y* is the envelope spectrum and $\vec{t}$ is the target vector.

Recognizing that rolling bearings commonly exhibit faults in the outer race, inner race, rolling elements, and so on, the MKurt value for each IMF is evaluated under these typical fault scenarios. This targeted analysis enables the identification of IMFs that are most relevant to specific fault types. An IMF is considered to contain meaningful fault-related features if its MKurt exceeds a certain threshold under a given condition. Once the relevant IMFs are identified, those corresponding to the same fault type are merged to consolidate diagnostic information, suppress redundancy, and improve feature clarity in the envelope spectrum.

In this study, a threshold of 1 × 10^−3^ is adopted based on extensive simulation and experimental results, as it offers a practical balance between sensitivity to weak fault impulses and robustness against noise interference. Importantly, this threshold is not fixed and can be adjusted according to the characteristics of the signal, such as noise level, fault type, and severity. A threshold that is too low may lead to the retention of noise-dominated components, while a threshold that is too high risks discarding weak yet diagnostically valuable features. Therefore, the selected value serves as a reliable default under typical conditions but can be flexibly adapted in other application scenarios as needed.

### 4.3. The Flowchart of Proposed IVMD for Bearing Fault Diagnosis

**Figure 5:**
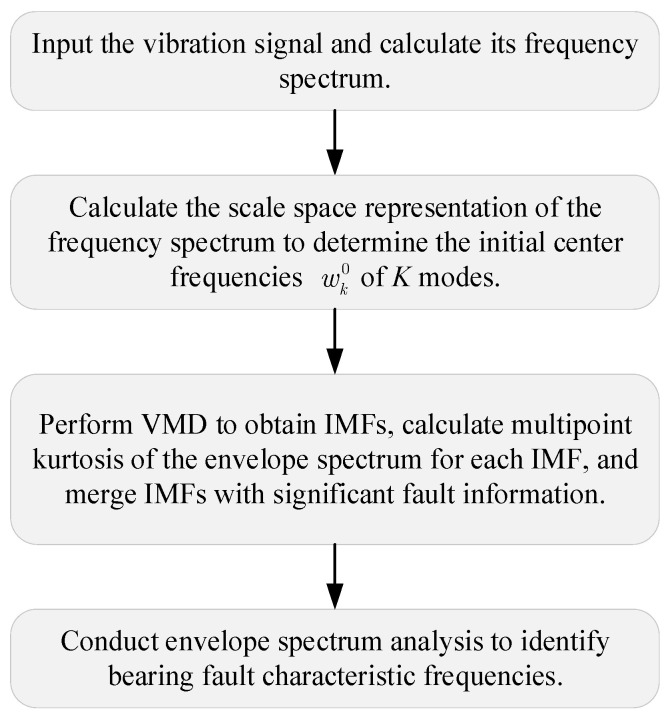
The flowchart of the proposed method for bearing fault diagnosis.

The proposed IVMD for bearing fault diagnosis is structured to systematically extract and analyze fault-related features from vibration signals. The flowchart, as depicted in Figure 5, encompasses the following detailed steps:

Step 1: Vibration signal acquisition and frequency spectrum computation

Acquire the vibration signal and compute its frequency spectrum via DFT to obtain the spectral distribution of the signal.

Step 2: Scale space representation for initialization

Apply scale space representation to the frequency spectrum to determine the initial center frequencies $w_{k}^{0}$ of *K* modes for VMD by detecting local maxima. This data-driven initialization improves the adaptivity and accuracy of mode decomposition.

Step 3: Variational mode decomposition and multipoint kurtosis analysis

Perform VMD using the initialized center frequencies. Compute the MKurt of the envelope spectrum for each IMF under the three common fault types (outer race, inner race, rolling elements). Identify IMFs whose MKurt exceeds the threshold and merge them based on fault type to reduce redundancy.

Step 4: Envelope spectrum analysis for fault feature identification

Conduct envelope analysis on the merged IMFs to extract characteristic fault frequencies, enabling accurate identification of specific bearing defects.

## 5. Experimental Validation

**Figure 6:**
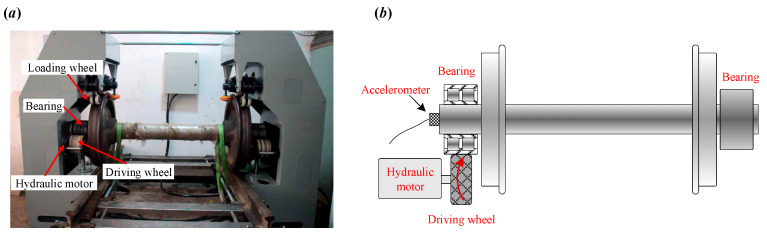
(**a**) Experiment setup of the test rig. (**b**) Schematic view of locomotive test rig.

To further verify the effectiveness of the proposed IVMD, an experimental study was conducted using a locomotive bearing test bed, as shown in Figure 6. The test platform consists of a driving wheel, a hydraulic motor, and a locomotive wheel. It is important to note that, during the experiment, the inner race of the bearing is fixed, while the outer race is rotated by the driving wheel. As a result, the fault impulses generated by an outer race fault are modulated, and corresponding sidebands are expected to appear in the envelope spectrum. In contrast, fault impulses caused by an inner race fault are not modulated and thus do not exhibit sidebands in the envelope spectrum. A tri-axial PCB accelerometer with a sensitivity of 100 mV/g was mounted at the end of the wheel axle to collect vibration signals, while a tachometer was used to record the rotational speed of the driving wheel. Both vibration and tachometer signals were collected over a 1.5 s period, with a sampling frequency of 76,800 Hz. In the experiment, the bearing under test is a tapered roller bearing, model 197,726. The bearing contains 20 rollers, with a pitch diameter of 180 mm and a roller diameter of 23.775 mm. The contact angle is 9 degrees, which influences the distribution of loads across the bearing elements. Based on these parameters, the fault characteristic frequencies can be calculated. To evaluate the advantages of the proposed IVMD, a comparison was made with the conventional VMD. Both methods were applied to the same dataset to assess performance differences.

### 5.1. Case 1: Bearing with an Inner Fault

To evaluate the effectiveness of the proposed IVMD, a vibration signal from a rolling element bearing with an inner race fault is analyzed. The bearing’s characteristic frequencies, including the rotation frequency (*f_r_*), the outer race fault frequency (BPFO), the inner race fault frequency (BPFI), and the ball spin frequency (BSF), are summarized in Table 2.

**Figure 7:**
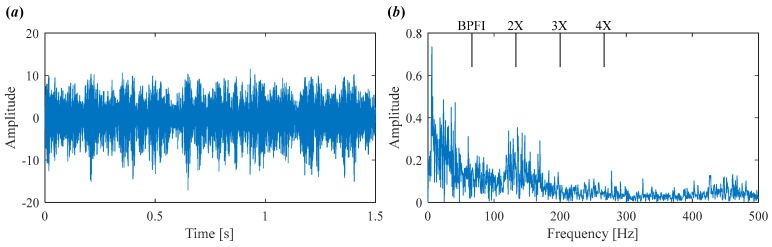
Vibration signal in Case 1. (**a**) Time domain waveform. (**b**) Envelope spectrum.

As shown in Figure 7a, the time-domain waveform is heavily contaminated by noise, making it difficult to observe any clear defect-related impulses. The corresponding envelope spectrum in Figure 7b also fails to provide useful fault information.

**Figure 8:**
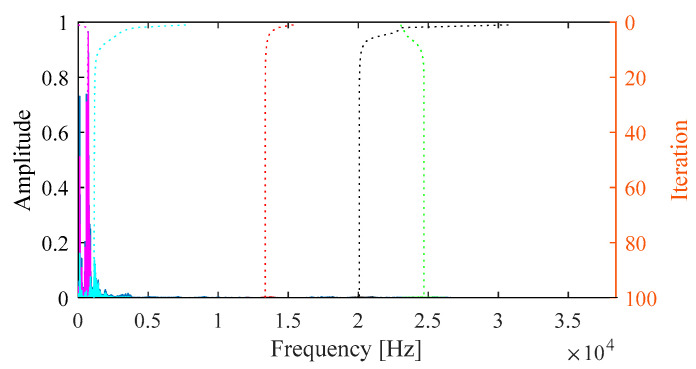
Iterative process of center frequencies in VMD.

**Figure 9:**
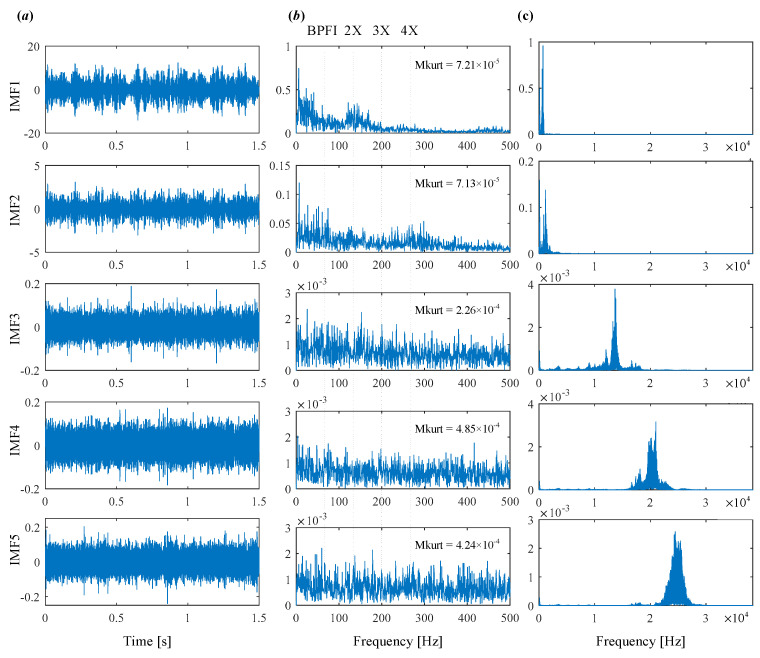
The modes obtained by VMD. (**a**) Time domain waveform. (**b**) Envelope spectrum. (**c**) Frequency spectrum.

To begin, the conventional VMD is applied to the signal. Figure 8 illustrates the iterative process of center frequency estimation and the resulting decomposition. In VMD, the mode number *K* and quadratic penalty term *α* for conventional VMD are set as 5 and 2000, respectively. Figure 9 presents the extracted modes along with their time-domain waveforms, envelope spectrum, and frequency spectrum. According to the selection metric Mkurt, none of the modes exhibit a value greater than 1 × 10^−3^, and no fault characteristic frequencies are observed in the envelope spectrum. This outcome indicates that the conventional VMD fails to identify the inner race fault due to inappropriate parameter settings.

**Figure 10:**
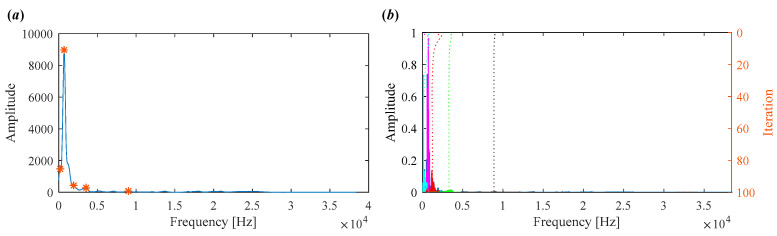
(**a**) Scale space representation and initial center frequencies. (**b**) Iterative process of center frequencies in IVMD.

For comparison, the proposed IVMD is applied to the same vibration signal. Figure 10a displays the scale space representation of the frequency spectrum, where the initial center frequencies of five modes are easily identified via peak detection. After determining the center frequencies and selecting the number of modes, the signal is decomposed. Figure 10b shows the iterative evolution of the center frequencies and the corresponding decomposition results.

**Figure 11:**
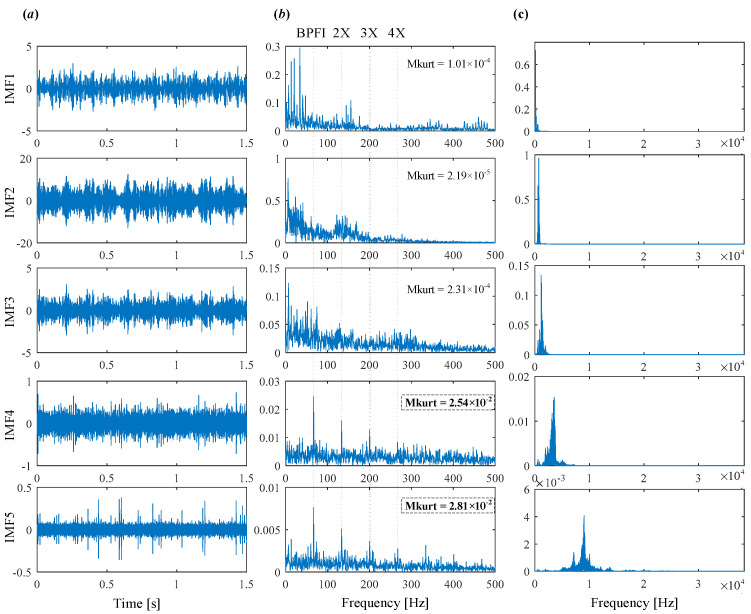
The modes obtained by IVMD. (**a**) Time domain waveform. (**b**) Envelope spectrum. (**c**) frequency spectrum.

**Figure 12:**
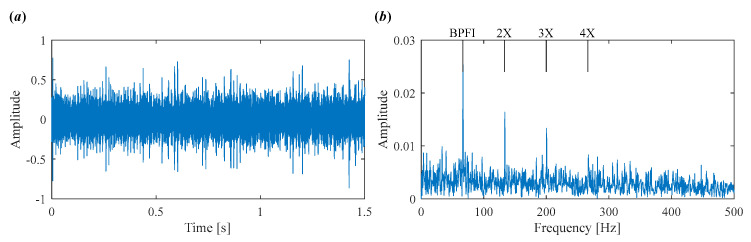
The results of IVMD after merging. (**a**) Time domain waveform. (**b**) Envelope spectrum.

**Figure 13:**
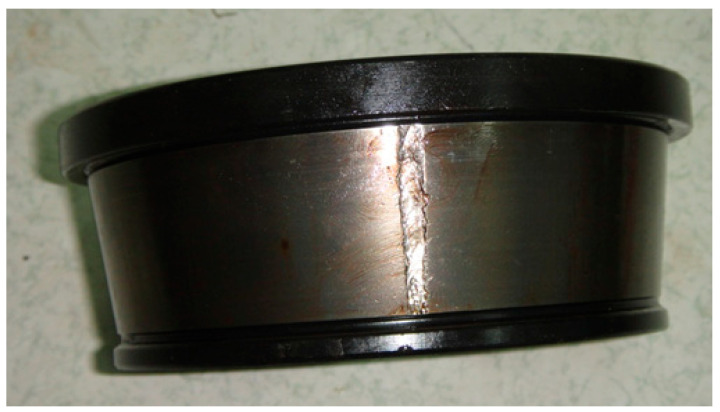
The defect on the inner race.

The decomposed modes obtained by IVMD are presented in Figure 11, including their time-domain waveforms, envelope spectrum, and frequency spectrum. It can be clearly seen that the fault characteristic frequency associated with the inner race is prominent in the envelope spectrum of IMF4 and IMF5. The corresponding Mkurt values for these modes exceed the threshold, providing strong evidence of an inner race fault. To further enhance the fault information, IMF4 and IMF5 that both exceed the Mkurt threshold are merged. The merged results are shown in Figure 12. As illustrated in Figure 12, the inner race fault frequency is clearly identified, and periodic defect impulses are visibly enhanced. Importantly, the sole distinction between the conventional VMD and the proposed IVMD lies in the initialization of center frequencies; all other parameters remained constant. The substantial improvement in performance underscores the critical role of appropriate center frequency initialization. The IVMD method adaptively determines these frequencies, facilitating optimal decomposition outcomes. Finally, to verify the diagnostic conclusion, the bearing is dismantled and the inner race is inspected. As shown in Figure 13, a spall fault is observed, confirming the presence of an inner race defect. This result not only validates the accuracy of IVMD but also demonstrates its superiority over conventional VMD. Due to its adaptability and robustness, IVMD is better suited for real-world fault diagnosis applications.

### 5.2. Case 2: Bearing with Multiple Faults

In this case, a real locomotive bearing exhibiting both outer race and inner race faults is tested to further verify the effectiveness of the proposed IVMD method in multi-fault diagnosis. The bearing characteristic frequencies are listed in Table 3.

**Figure 14:**
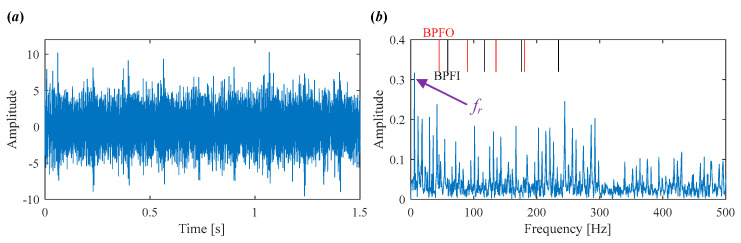
Vibration signal in Case 2. (**a**) Time domain waveform. (**b**) Envelope spectrum.

As shown in Figure 14a, the time-domain waveform of the original vibration signal is severely contaminated by noise and various interferences, making it difficult to detect any periodic impulses indicative of bearing faults. The envelope spectrum in Figure 14b is dominated by the rotating frequency *f_r_* and its harmonics, while the bearing fault characteristic frequencies are completely obscured.

**Figure 15:**
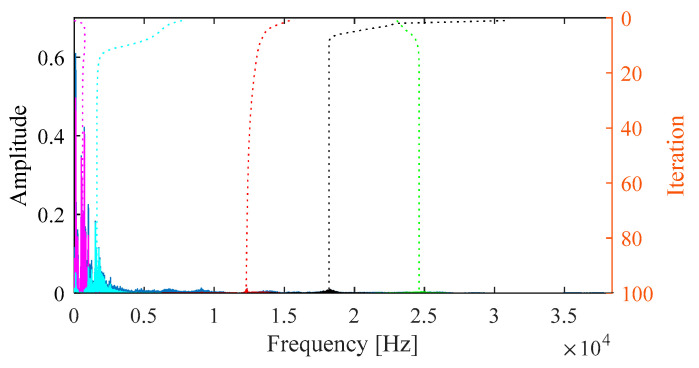
Iterative process of center frequencies in VMD.

**Figure 16:**
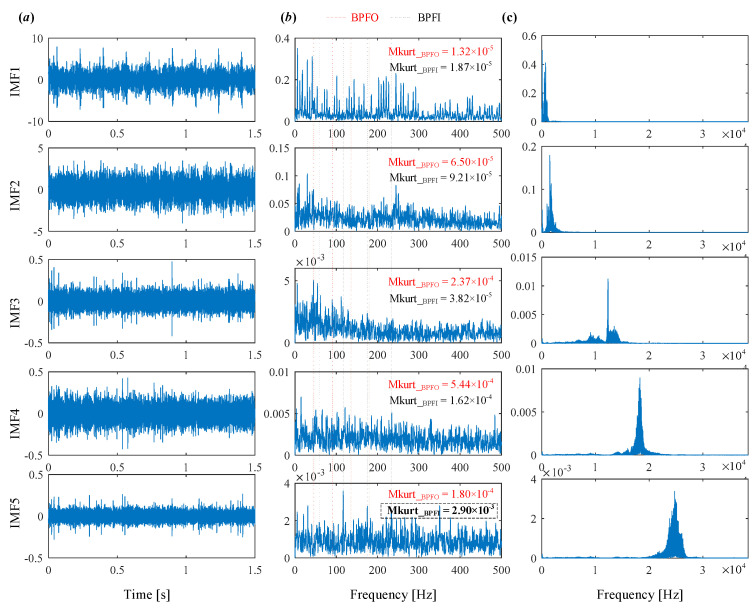
Modes obtained by VMD. (**a**) Time domain waveform. (**b**) Envelope spectrum. (**c**) Frequency spectrum.

The conventional VMD is first applied to analyze the raw signal, with results shown in Figure 15 and Figure 16. Figure 15 illustrates the iterative evolution of center frequencies, where the initialization is performed according to $P_{u}$ distribution. Figure 16 presents the extracted modes, including their time-domain waveforms, envelope spectra, and frequency spectra. The Mkurt values for the envelope spectra of each mode are also calculated. As shown, only IMF5 has an Mkurt__BPFI_ value exceeding the threshold, indicating detection of the inner race fault. This is confirmed in Figure 16b, where spectral lines at 2×BPFI and 3×BPFI appear in the envelope spectrum of IMF5. However, no evidence of the outer race fault is found. Hence, the conventional VMD fails to identify the coexisting faults in this case, detecting only the inner race defect while completely missing the outer race fault.

**Figure 17:**
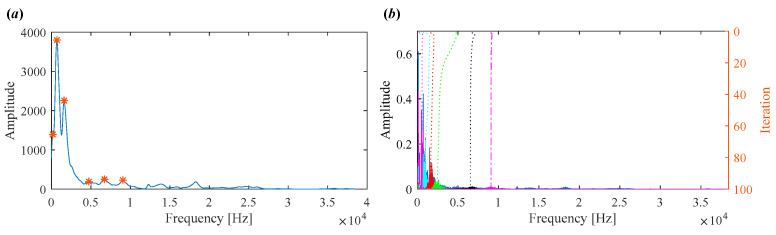
(**a**) Scale space representation and initial center frequencies. (**b**) Iterative process of center frequencies in IVMD.

**Figure 18:**
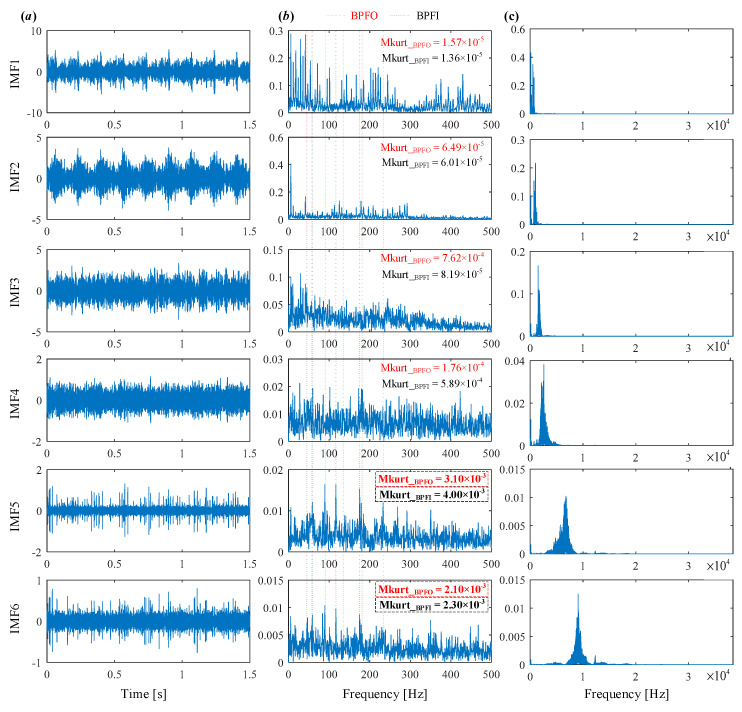
Modes obtained by IVMD. (**a**) Time domain waveform. (**b**) Envelope spectrum. (**c**) Frequency spectrum.

**Figure 19:**
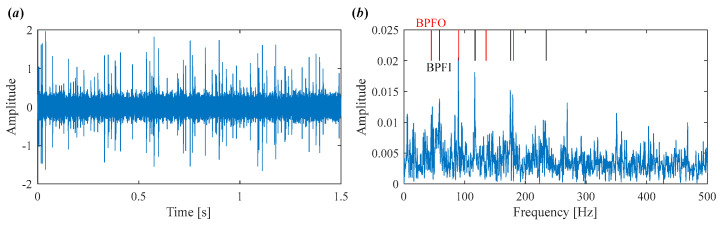
The final results of IVMD after merging. (**a**) Time domain waveform. (**b**) Envelope spectrum.

**Figure 20:**
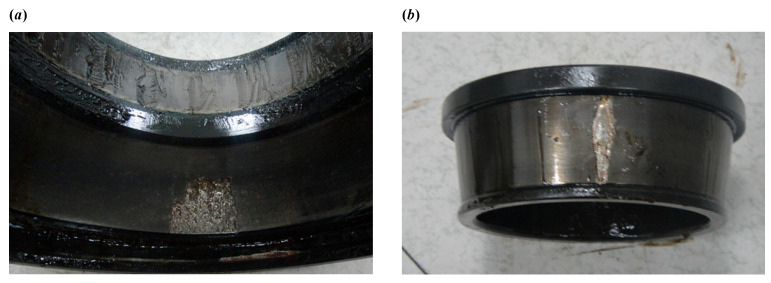
(**a**) The defect on the outer race. (**b**) The defect on the inner race.

To demonstrate the capability of the proposed IVMD in extracting weak fault features, the same signal is analyzed using IVMD. The results are displayed in Figure 17 and Figure 18. Figure 17a shows the scale space representation of the frequency spectrum, from which initial center frequencies are determined through peak detection and then sequentially applied during decomposition. The iterative adjustment of center frequencies is shown in Figure 17b. All modes extracted via IVMD are presented in Figure 18, along with their Mkurt values. It is evident from Figure 18b that both IMF5 and IMF6 contain significant information related to the inner and outer race faults. Specifically, the Mkurt values corresponding to both BPFO and BPFI exceed the threshold in these modes. Moreover, the fault characteristic frequencies are clearly observable in their envelope spectrum, demonstrating the effectiveness of IVMD in identifying multiple coexisting faults. In practical diagnosis, only the merged result of the selected modes is used, while the presentation of all modes in the paper serves to enhance clarity. As shown in Figure 19b, the merged result distinctly reveals fault-related spectral lines at 2×BPFO, 4×BPFO, 2×BPFI, and 3×BPFI, indicating the simultaneous presence of outer and inner race faults. Additionally, periodic fault impulses are clearly observed in the time-domain waveform. Compared to the conventional VMD, the proposed IVMD exhibits significant superiority in multi-fault diagnosis due to its adaptive center frequency initialization and more accurate mode decomposition. Finally, the bearing was disassembled for physical inspection. Figure 20 provides photographic evidence of spall defects on both the outer and inner raceways, which confirms the diagnosis results obtained via IVMD and further demonstrates the method’s robustness and reliability in practical applications.

## 6. Conclusions

This study proposes IVMD to adaptively determine the initial center frequencies and mode number based on scale space representation. By applying a peak search within the frequency spectrum, IVMD enables a data-driven and more reasonable parameter initialization. The signal is subsequently decomposed, and the resulting modes are evaluated using the MKurt index. Modes with MKurt values exceeding the threshold are merged to enhance the fault information, thereby facilitating the precise identification of fault characteristic frequencies in the envelope spectrum. Experimental results demonstrate that IVMD significantly improves the accuracy and reliability of fault feature extraction compared to conventional VMD, especially under noisy conditions. The method exhibits strong adaptability and robustness, effectively addressing the challenges of weak fault signal detection in practical scenarios. Furthermore, the successful diagnosis of both single and multiple faults validates the capability of IVMD in complex fault diagnosis tasks. Overall, IVMD provides a practical, data-driven solution for bearing fault diagnosis and can be extended to other rotating machinery systems. Future research will focus on real-time implementation, integration with intelligent classifiers for automated fault identification, and validation under variable operating conditions.

## Figures and Tables

**Table 1:** The parameters of the simulated signal.

Rotor or Shaft						Outer Race Fault				Inner Race Fault			
*f*_1_	*A*_1_	*α*_1_	*f*_2_	*A*_2_	*α*_2_	*D*_1_	*T*_1_	*f*_r1_	*ξ*_1_	*D*_2_	*T*_2_	*f*_r2_	*ξ*_2_
10	0.4	π/3	20	0.25	π/6	1.3	1/27	1500	300	1	1/50	3200	400

**Table 2:** Bearing fault characteristic frequencies (Hz) in Case 1.

*f_r_*	BPFO	BPFI	BSF
5.8920	51.2331	66.6060	21.9243

**Table 3:** Bearing fault characteristic frequencies (Hz) in Case 2.

*f_r_*	BPFO	BPFI	BSF
5.1860	45.0949	58.6261	19.2976

## Data Availability

Data are unavailable due to privacy restrictions.
